# Architectural anatomy of the human tibialis anterior presents morphological asymmetries between superficial and deep unipennate regions

**DOI:** 10.1111/joa.13864

**Published:** 2023-03-30

**Authors:** Saúl Martin‐Rodriguez, Juan Jose Gonzalez‐Henriquez, Victor Galvan‐Alvarez, Sara Cruz‐Ramírez, José A. Calbet, Joaquín Sanchis‐Moysi

**Affiliations:** ^1^ Department of Physical Education University of Las Palmas de Gran Canaria Las Palmas de Gran Canaria Spain; ^2^ Research Institute of Biomedical and Health Sciences (IUIBS) Las Palmas de Gran Canaria Spain; ^3^ Department of Mathematics University of Las Palmas de Gran Canaria Las Palmas de Gran Canaria Spain; ^4^ Department of Physical Performance The Norwegian School of Sport Sciences Oslo Norway

**Keywords:** fascicle length, muscle architecture, muscle thickness, pennation angle, tibialis anterior

## Abstract

The tibialis anterior muscle plays a critical role in human ambulation and contributes to maintaining the upright posture. However, little is known about its muscle architecture in males and females. One hundred and nine physically active males and females were recruited. Tibialis anterior muscle thickness, pennation angle, and fascicle length were measured at rest in both unipennate regions of both legs using real‐time ultrasound imaging. A linear mixed model was used with muscle thickness, pennation angle, or fascicle length as the dependent variables. All models were carried out with and without total leg lean mass and shank length as covariates. Causal mediation analysis was computed to explore the effect of muscle thickness on the relationship between fascicle length and pennation angle. There were no significant differences between dominant and nondominant legs regarding muscle architecture. Muscle thickness and pennation angle were greater in the deep than the superficial unipennate region in males (1.9 mm and 1.1°, *p* < 0.001) and women (3.4 mm and 2.2°, *p* < 0.001). However, the fascicle length was similar in both regions for both sexes. The differences remained significant after accounting for differences in leg lean mass and shank length. In both regions, muscle thickness was 1–3 mm greater in males and superficial pennation angle 2° smaller in females (both, *p* < 0.001). After accounting for leg lean mass and shank length, sex differences remained for muscle thickness (1.6 mm, *p* < 0.05) and pennation angle (3.4°, *p* < 0.001) but only in the superficial region. In both regions, leg lean mass and shank‐adjusted fascicle length were 1.4 mm longer in females than males (*p* < 0.05). The causal mediation analysis revealed that the estimation of fascicle length was positive, suggesting that a 10% increase in muscle thickness would augment the fascicle length, allowing a 0.38° pennation angle decrease. Moreover, the pennation angle increases in total by 0.54° due to the suppressive effect of the increase in fascicle length. The estimated mediation, direct, and total effects were all significantly different from zero (*p* < 0.001). Overall, our results indicate that the architectural anatomy of the tibialis anterior shows sexual dimorphism in humans. Tibialis anterior presents morphological asymmetries between superficial and deep unipennate regions in both sexes. Lastly, our causal mediation model identified a suppressive effect of fascicle length on the pennation angle, suggesting that increments in muscle thickness are not always aligned with increments in fascicle length or the pennation angle.

## Graphical abstract

For more than 20 years, it has been considered that the tibialis anterior was a bipennate muscle whose unipennate regions behaved like a mirror, that is, their architectural characteristics were similar at each side of the central aponeurosis. Our results indicate that the architectural anatomy of the tibialis anterior shows sexual dimorphism in humans. Moreover, the tibialis anterior presents morphological asymmetries between superficial and deep unipennate regions in both sexes.

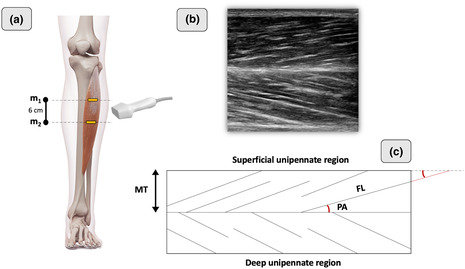

## 1 INTRODUCTION

The human tibialis anterior (TA) is the largest muscle in the anterior compartment of the lower leg and accounts for over 60% of the ankle dorsiflexor muscle volume, sharing functions with the extensor hallucis longus, and the extensor digitorum longus (Keith et al., 2006). The main role of the TA is the dorsiflexion and inversion of the foot (Keith et al., 2006). The TA contributes to maintaining the upright posture (Di Giulio et al., 2009) and plays a key role in energy absorption during walking (Maharaj et al., 2019). Consequently, motor disorders affecting TA size and strength, such as cerebral palsy, negatively impact the gait cycle (Bland et al., 2011). Moreover, the age‐associated decline in muscle strength of the TA has been found to increase the risk of falls (Perry et al., 2007).

Anatomically, the TA muscle originates on the lateral condyle of the tibia, on the upper two‐thirds of the lateral surface of this bone, on the anterior surface of the interosseous membrane, and on the deep surface of the fascia cruris (Keith et al., 2006). On the other end, the distal attachment is typically at the medial cuneiform and first metatarsal bones (Zielinska et al., 2021). From a muscular architectural point of view, the TA is a bipennate muscle (Alexander, 1975) with a superficial and a deep region (Maganaris & Baltzopoulos, 1999), which have been considered symmetrical (Maganaris & Baltzopoulos, 1999) based on the ultrasound analysis of six men. To the best of our knowledge, the study by Maganaris and Baltzopoulos (1999) is unique and has not been replicated. Moreover, it remains unknown whether sex differences exist in the anatomical architecture of the TA in humans.

The architecture of a muscle has important functional consequences. For a given muscle volume, parallel‐fibered muscles can generate larger excursions and achieve faster shortening speeds because they have more sarcomeres in series (Eng et al., 2018). In contrast, pennate muscles (e.g., TA) allow for more parallel sarcomeres, leading to large forces for a given muscle length (Powell et al., 1984). The main muscle architecture features are muscle thickness (MT), cross‐sectional area, pennation angle (PA), and fascicle length (FL) (Eng et al., 2018). Pennation angles can vary within a muscle, and this influences local fiber strains and hence, gearing, within a muscle (Azizi & Deslauriers, 2014). The simple geometric model used by Azizi and Deslauriers (2014) predicts that fibers with a lower PA undergo greater fiber strains than the more pennate fibers and that this difference will increase with the magnitude of the muscle strain. This means that under most conditions, fibers with a higher PA work at a higher gear ratio than fibers with a lower PA. However, the relationship between PA with MT, and FL is not always direct or causal. In this regard, discrepancies have been found in the literature when MT and PA are modified after resistance training but not FL (Franchi et al., 2016; Fukutani & Kurihara, 2015). The relationship between MT, PA, and FL could be studied by using causal mediation analysis (Nuzzo et al., 2019), although this technique has not been applied to the TA.

Increased knowledge of the human TA muscle architecture will provide fundamental anatomical information, which may be useful in the clinical setting and to develop specific programs for rehabilitation or to increase sports performance. Based on these previous findings, we hypothesized that superficial and deep unipennate regions of the TA would be symmetrical. Second, we hypothesized that there would be muscle architecture differences in the TA attending to sex variables. Lastly, we hypothesized that FL would have a direct effect on the relationship between MT and PA. This study aimed to test these hypotheses.

## 2 METHODS

### 2.1 Study design and participants

This is a cross‐sectional study comprising two separate measurement sessions. The first measurement session was conducted to perform pretests: anthropometrics and dual‐energy X‐ray absorptiometry (Lunar iDXA, General Electric) whole‐body scans, as previously reported (Calbet et al., 2017). Besides, as a part of the anthropometric measurements, the shank length (i.e., the distance between the proximal head of the fibula to the tip of the lateral malleolus) of both legs was measured as previously reported (Kunimasa et al., 2014). In a second visit, the participant's TA was explored by ultrasound. One hundred nine physically active and healthy males and females volunteered to participate in the study (Table 1). Subjects exercised regularly, performing between 3 and 8 h of mostly moderate‐intensity physical activity per week. Several subjects had a diverse sports trajectory, with participation in different sports throughout their career, while most of them had practiced soccer during part of their career. The inclusion criteria for participation in this investigation were: age 18–35 years; no chronic diseases or recent surgery; nonsmoking; normal resting electrocardiogram; body mass index above 18 and below 30; no medical contraindications to exercise; and no history of disease requiring medical treatments lasting longer than 15 days during the preceding 6 months. All volunteers signed a written consent after receiving information about the aims and potential risks of the study. The study commenced after approval by the Ethical Committee of the University of Las Palmas de Gran Canaria (CEIH2017/13) and was carried out according to the Declaration of Helsinki. The sex and gender of the participants were defined based on self‐reports during participant recruitment, and all participants reported *cis* gender.

**TABLE 1:** Descriptive characteristics of the study population reported as mean and between bracket the standard deviation.

Variable	Males (*n* = 64)	Females (*n* = 45)	All (*n* = 109)
Age (years)	23.1 (3.2)	23.0 (2.8)	23.0 (3.0)
Height (cm)	176.7 (6.8)	164.5 (5.9)^†^	171.7 (8.8)
Weight (kg)	74.2 (7.4)	59.4 (8.5)^†^	68.1 (10.7)
BMI (kg/m^2^)	23.8 (2.2)	21.9 (2.7)^†^	23.0 (2.6)
Total BM (g)	3175.3 (332.1)	2444.7 (276.4)^†^	2873.7 (475.3)
Total FM (g)	14346.4 (4486.6)	16481.1 (4527.3)^†^	15227.7 (4614.8)
Total LM (g)	56633.7 (5045.0)	40461.1 (5276.8)^†^	49957.0 (9487.3)
Left leg LM (g)	9977.2 (909.5)	6484.2 (927.3)^†^	9301.2 (1659.9)
Right leg LM (g)	10148.4 (935.5)	6508.5 (946.2)^†^	9443.9 (1744.5)
Both legs LM (g)	20233.4 (1948.3)	14220.7 (2079.6)^†^	17751.1 (3577.6)
Fat (%)	19.1 (4.7)	27.5 (4.7)^†^	22.6 (6.3)
Right dominant leg (%)	0.80 (0.4)	0.96 (0.2)^†^	0.86 (0.4)
Shank length (cm)	37.8 (2.36)	34.5 (1.80)^†^	36.7 (2.69)
Superficial TA Thickness (cm)	1.23 (0.19)	0.94 (0.17)^†^	1.11 (0.23)
Deep TA thickness (cm)	1.41 (0.22)	1.28 (0.19)^†^	1.36 (0.21)
Total TA thickness (cm)	2.64 (0.30)	2.23 (0.25)^†^	2.47 (0.35)
Superficial TA FL (cm)	6.41 (1.44)	6.46 (1.79)	6.43 (1.59)
Deep TA FL (cm)	6.55 (1.61)	6.52 (1.41)	6.54 (1.53)
Superficial TA PA (°)	11.62 (2.53)	9.52 (2.45)^†^	10.75 (2.70)
Deep TA PA (°)	12.76 (2.65)	11.74 (3.14)^‡^	12.34 (2.90)

*Note*: The shank length and all architectural variables are described as averaged values of both legs. *p‐*values presented correspond to comparisons between males and females (^†^ *p* < 0.001; ^‡^ *p* < 0.02).

Abbreviations: BM, bone mass; FL, fascicle length; FM, fat mass; LM, lean mass; PA, pennation angle; TA, tibialis anterior.

### 2.2 Ultrasound imaging

**FIGURE 1:**
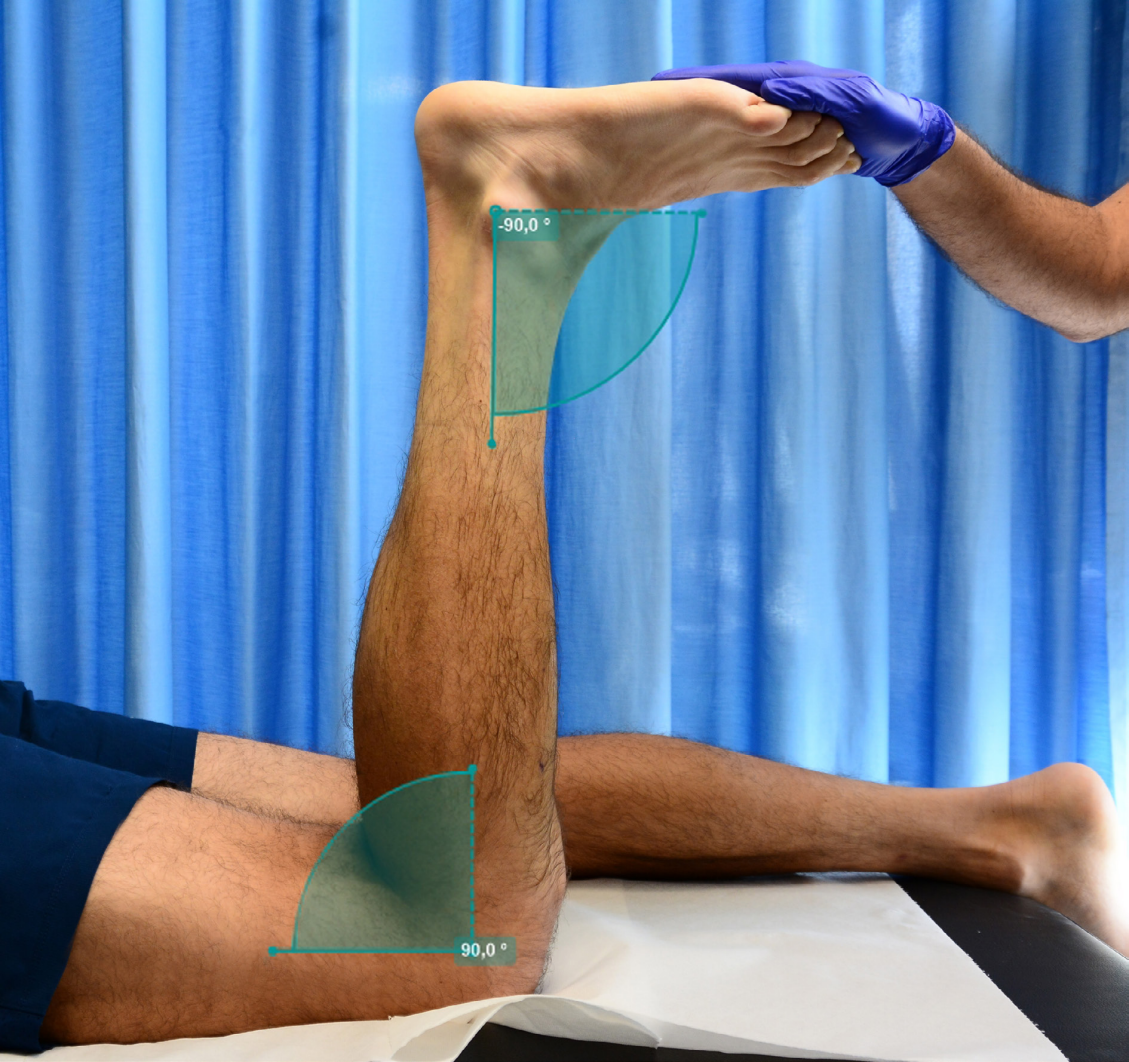
Measurement setup. The knee and ankle joint angles were set at 90°, and the angle was verified with a manual goniometer.

**FIGURE 2:**
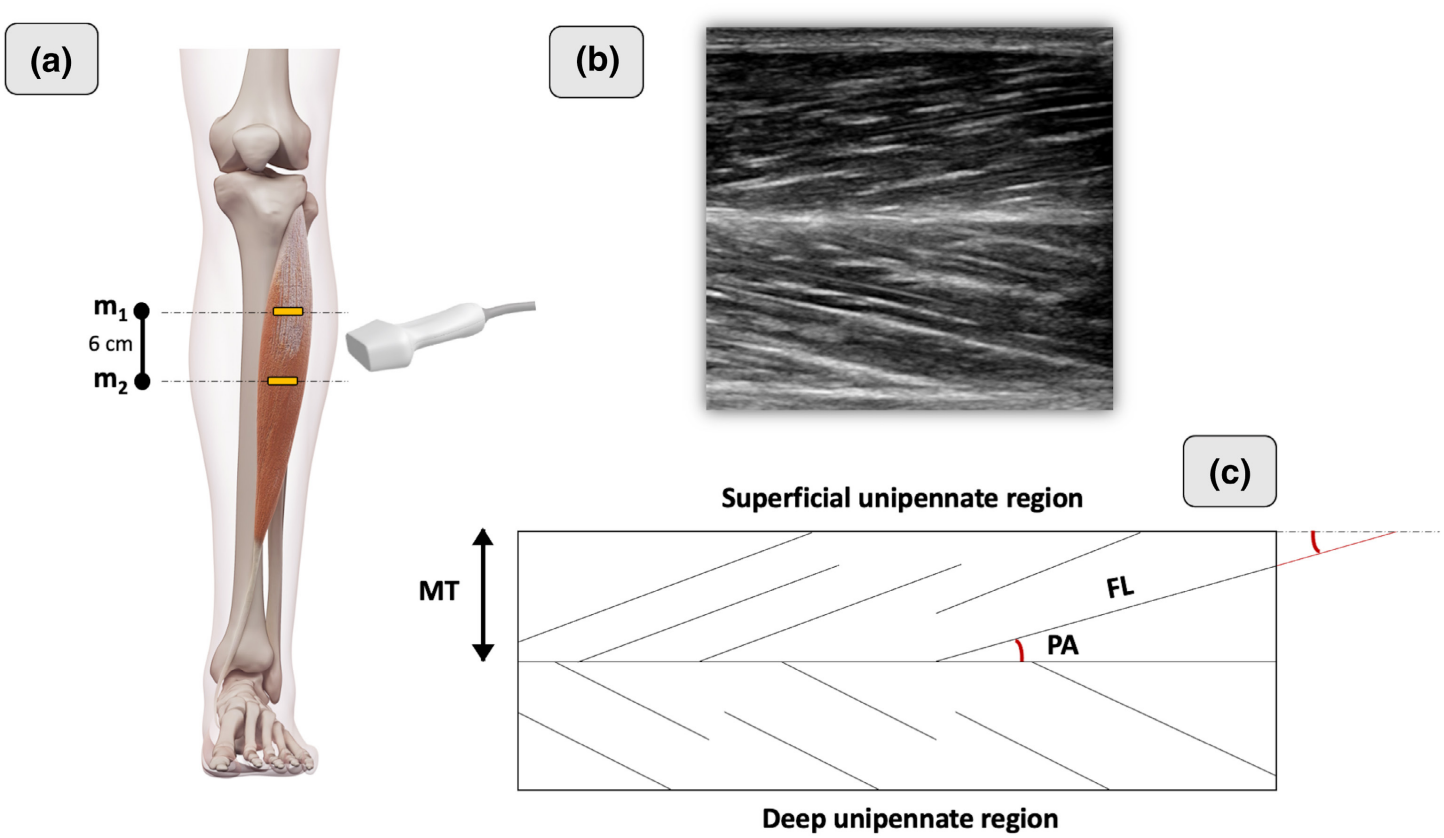
Ultrasound imaging protocol used in both unipennate regions of the TA. (a) The proximal margin of the TA central aponeurosis was determined from the sagittal images of the TA and was marked on the dermal surface (m_1_), then the measurement site was placed 6 cm below the proximal end of the central TA aponeurosis (m_2_) (b) Representative image of the TA and its two unipennate regions (c) Planimetric model of the TA indicating that FL was calculated by linear extrapolation of the visible portion of fascicles to the intersection point with the linearly projected superficial aponeurosis of the muscle. FL, fascicle length; MT, muscle thickness; PA, pennation angle; TA, tibialis anterior.

Real‐time two‐dimensional B‐mode ultrasound (Philips CX50, Philips Medical Systems, Netherlands) with a 38 mm linear‐array transducer (12–3 MHz, L12‐3 Broadband, Phillips), was used to bilaterally measure the muscle architecture of the TA. An operator with extensive experience in musculoskeletal ultrasonography performed image acquisition. Current guidelines and recommendations for musculoskeletal ultrasound by the European Federation of Societies for Ultrasound in Medicine and Biology were followed (Fodor et al., 2022). The ultrasound depth was adjusted to 4–5 cm depending on the individual participant and the frequency was adjusted between 38–41 Hz. The probe was hand‐held, and the measurements were made with the subject in a prone position with the knee flexed at 90° (Maganaris & Baltzopoulos, 1999) while the ankle was kept at 90° (Figure 1). Knee and ankle angles were checked using a manual goniometer. The ultrasound probe was placed perpendicular to the skin, and a water‐soluble gel was applied to the skin to obtain a high‐resolution image without losing the detailed anatomical features of the muscles (Ihnatsenka & Boezaart, 2010). Each measurement site was marked on the skin surface with a surgical pen to ensure that the probe was placed in the proper position. The use of the gel meant that the ultrasound probe could be positioned just above the skin surface at each landmark without pressure being applied to the skin. The proximal margin of the TA central aponeurosis was determined from the sagittal images of the TA and was marked on the dermal surface. The measurement site was placed 6 cm below the proximal end of the central TA aponeurosis (Muraoka et al., 2003) (Figure 2). The primary inclusion criterion for ultrasound image analyses was that the aponeuroses were parallel as the angle between the superficial and the intermediate aponeuroses can strongly influence the extrapolation methodologies (Blazevich et al., 2006; Franchi et al., 2018).

### 2.3 Tibialis anterior architecture assessment

In each unipennate region of the TA, the PA was measured at the fascicle insertions into the superficial (or deep) and central aponeuroses. MT in each unipennate region was measured as the distance between the superficial (or deep) and central aponeuroses in both images' ends. Since TA fascicles were longer than the width of the probe, fascicle length was calculated by linear extrapolation of the visible portion of fascicles to the intersection point with the linearly projected superficial aponeurosis of the muscle (Potier et al., 2009). In total, 6 PAs corresponding to 3 FLs were analyzed in each unipennate region, i.e., 3 PA corresponding to the superficial and 3 PA corresponding to the central aponeuroses of each unipennate region following previously described procedures to assess TA muscle architecture (Maganaris & Baltzopoulos, 1999). The inclusion criteria for determining appropriate fascicles to analyze were the following: the fascicle insertion point into the central (or deep) aponeurosis must have been visible, and a reasonable portion of the fascicle (~25% or more of the total estimated length) must have been visible within the ultrasound transducer's field of view (Franchi et al., 2020). Muscle architectonic parameters (MT, PA, and FL) were digitized using image‐processing software (OsiriX™ DICOM viewer, Pixmeo). Overall, 218 images and almost ~5000 measures (12 measures per leg) were recorded in all participants. Ultrasound reliability was tested in four males before the start of the study. In brief, the operator acquired one image of the TA of each male at rest in the morning, in a relaxed state and without having exercised or done any vigorous activity in the previous 72 h. A person other than the operator segmented the images taken that day, without knowing to whom each image belonged, that is, the images were blinded. This same procedure was performed 3 days later. Thus, the intraclass correlation coefficient (ICC 3.1) was 0.89 for MT, 0.88 for PA, and 0.78 for FL. Our intrarater reliability is in line with the literature (Kwah et al., 2013) and it has been described according to a reference guideline for selecting and reporting for reliability research (Koo & Li, 2016).

### 2.4 Variables for data analysis

Leg dominance (dominant vs. nondominant), muscle architectural features (MT, PA, and FL), unipennate region (superficial vs. deep), sex, LLM, and shank length were the main variables of data analysis.

### 2.5 Statistical analysis

Descriptive data are presented as the mean and standard deviation (SD). Males' and females' general characteristics were compared using an unpaired t‐test. A linear mixed model with MT as the dependent variable, fixed effects (sex, leg dominance, both unipennate regions, and the interaction), and random intercept (subjects) was applied to compare both unipennate regions. This linear mixed model introduced lower extremities lean mass (LLM) and shank length as covariates. The same approach was adopted for FL and PA. For all models, marginal means, and standard errors (SEM) were estimated for all factor combinations. Averaged PAs (average of 6 PAs in each unipennate region) and FLs (average of 3 FLs in each unipennate region) values were used for all models to characterize each unipennate region.

A posteriori*‐planned* analysis consisted of exploring the effect of MT on the relationship between FL and PA. Mediation analysis for mixed models was carried out since this study has three variables (i.e., MT, FL, and PA) measured in the same participant in four different conditions, i.e., two factors within participants with two levels: leg dominance (dominant vs. nondominant) and unipennate region (superficial or deep). In the mediator model, FL (i.e., the mediator) was modeled as a linear mixed model, with MT adjusted by the dominant leg, and each unipennate region, sex, and their interaction as predictors. The outcome model, also a linear mixed model with PA as the dependent variable, included MT, the mediator (i.e., FL), and the same set of variables used in the mediator model as explanatory variables. Both the mediator and outcome models have random intercepts (subjects). Since MT is a continuous variable, it is necessary to set two MT values to estimate the effect that this change in MT has on the PA, both directly and indirectly through FL (Imai et al., 2010). The increment in MT was set at 10%. Our mediation analyses are in line with the AGReMA statement (A Guideline for Reporting Mediation Analyses) of randomized controlled trials and observational studies (Lee et al., 2021). The corresponding AGReMA checklist is provided as Data S1.

All statistical analyses were conducted with R 4.2.2 (R Foundation for Statistical Computing, Vienna, Austria). The package “emmeans” for R was used to estimate marginal means (Lenth et al., 2018). The mediation analysis was carried out using the R package “mediation” (Tingley et al., 2014). Given the number of participants included in this study (*n* = 109), we applied the central limit theorem and considered that the data could be analyzed with parametrical statistics. Statistical significance was set at *p* < 0.05.

## 3 RESULTS

### 3.1 Study population

This study analyzed one hundred and nine participants from 20 to 26 years old. The descriptive characteristics of the study population are reported in Table 1. Females had a higher body fat percentage, less total lean mass, and less total leg lean mass than men. Most of the participants were right‐legged. Significant differences were found in muscle architecture characteristics (i.e., MT and PA) of the TA between males and females, except for the FL of the superficial and deep unipennate regions. Males had a 3.3 cm longer shank length than females (*p* < 0.001), while no significant differences were observed between the dominant and nondominant legs in both sexes (Table 1).

### 3.2 Muscle architecture: Main findings and sex differences

Linear mixed model results are shown in Table 2. There were no significant differences between dominant and nondominant legs regarding muscle architecture. MT and PA were significantly greater in the deep than the superficial unipennate region in men (1.9 mm and 1.1°, *p* < 0.001) and women (3.4 mm and 2.2°, *p* < 0.001). However, the FL was similar in both regions for both sexes. The differences remained significant after accounting for differences in LLM and shank length. Compared with females, the superficial and deep regions of the TA were 1.3 and 2.8 mm thicker in men (*p* < 0.001), and superficial PA was 2.1° greater (*p* < 0.001). After accounting for LLM and shank length, sex differences remained for MT (1.6 mm, *p* = 0.02) and PA (3.4°, *p* < 0.001) in the superficial region. In both regions, LLM and shank‐adjusted FL was 1.4 mm longer in females than males (*p* < 0.05) (Table **2**).

**TABLE 2:** Linear mixed model results.

Unipennate region	Males			Females			Between sex differences		
	MT (cm)	FL (cm)	PA (°)	MT (cm)	FL (cm)	PA (°)	MT (cm)	FL (cm)	PA (°)
Non‐adjusted model									
Superficial	1.23 (0.02)	6.41 (0.16)	11.62 (0.28)	0.94 (0.02)	6.46 (0.19)	9.52 (0.34)	**0.28 (0.03)** ^**†**^	−0.05 (0.25)	**2.10 (0.44)** ^**†**^
Deep	1.41 (0.02)	6.55 (0.16)	12.74 (0.28)	1.28 (0.02)	6.52 (0.20)	11.72 (0.34)	**0.13 (0.03)** ^**†**^	0.03 (0.25)	1.03 (0.44)
Difference	**0.19 (0.02)** ^**†**^	0.14 (0.16)	**1.12 (0.26)** ^**†**^	**0.34 (0.02)** ^**†**^	0.06 (0.19)	**2.20 (0.31)** ^**†**^	–	–	–
Adjusted model									
Superficial	1.19 (0.03)	5.99 (0.22)	12.03 (0.40)	1.03 (0.04)	7.38 (0.33)	8.65 (0.62)	**0.16 (0.05)** ^**‡**^	**−1.39 (0.47)** ^**‡**^	**3.39 (0.88)** ^**†**^
Deep	1.34 (0.03)	6.04 (0.22)	13.01 (0.40)	1.40 (0.04)	7.46 (0.33)	11.32 (0.62)	−0.06 (0.05)	**−1.43 (0.47)** ^**§**^	1.69 (0.87)
Difference	**0.15 (0.02)** ^**†**^	0.05 (0.17)	**0.98 (0.29)** ^**†**^	**0.37 (0.03)** ^**†**^	0.08 (0.23)	**2.67 (0.39)** ^**†**^	–	–	–

*Note*: Values are presented as the mean (SEM). Bonferroni‐adjusted pairwise comparisons were used (^†^ *p* < 0.001; ^‡^ *p* = 0.02; ^§^ *p* = 0.01). In the adjusted model, the marginal mean was estimated with LLM = 17,751 g (the overall mean LLM) and with shank length = 36.7 cm (the overall mean shank length).

Abbreviations: FL, fascicle length; MT, muscle thickness; PA, pennation angle.

### 3.3 Causal mediation analysis

The estimated mediation, direct, and total effects were all significantly different from zero (*p* < 0.001) (Table 3). In the mediation model, the estimation of the mediator coefficient (i.e., FL) was positive, suggesting that a 10% increase in MT would increase FL eliciting, through the increase in FL, a 0.38° reduction of the PA. Likewise, this 10% increase in MT has a direct effect of 0.92° on PA, implying that the PA increases by 0.92° per each 10% increase of MT, for a given FL. Since a higher thickness augments the FL, the PA increases in total by 0.54° due to the suppressive effect of the increase of FL.

**TABLE 3:** Causal mediation analysis on the effect of muscle thickness in the relationship between fascicle length and pennation angle.

Effect	Estimate	95% interval confidence
Indirect	−0.38*	(−50, −0.25)
Direct	0.92*	(0.83, 1.01)
Total	0.54*	(0.40, 0.70)

*Note*: Quasi‐Bayesian mediation analyses with one thousand simulations.

* *p* < 0.001.

## 4 DISCUSSION

The present investigation shows that TA muscle's superficial and deep unipennate regions are morphologically asymmetrical in males and females. In both sexes, the deep region displays higher MT (+2–4 mm) and greater PA (+1–2°) than the superficial region. These findings contrast with the symmetry reported by Maganaris and Baltzopoulos (Maganaris & Baltzopoulos, 1999) who did not find architectural differences between the superficial and deep portions of the TA in six males measured at rest and during a maximal voluntary contraction. We have also modeled how an increase in MT would affect FL and the PA.

### 4.1 The superficial and deep regions of the tibialis anterior muscle are not symmetrical

For more than 20 years, it has been considered that the TA was a bipennate muscle whose unipennate regions behaved like a mirror, that is, their architectural characteristics were similar at each side of the central aponeurosis. This is based on a pioneer study designed to assess the predictability of in vivo, ultrasound‐based changes in human TA pennation angle from rest to maximum isometric dorsiflexion in 6 men (Maganaris & Baltzopoulos, 1999). Using a planimetric model assuming constant thickness between superficial and deep aponeuroses and straight muscle fibers, the authors proposed that if the unipennate regions occupy equal volumes, each region accounts for half the force generated by the whole TA during a dorsiflexor MVC. In agreement with Maganaris and Baltzopoulos (Maganaris & Baltzopoulos, 1999), we observed similar FL in the superficial and deep unipennate regions of the TA. In contrast to Maganaris and Baltzopoulos (1999), we found that MT and PA are greater in the deep than in the superficial region. Our results are robust due to the use of multiple measurements in both legs, in a large sample size, minimizing type II error (Freiman et al., 2019).

Available published data on MT of human TA with similar participant characteristics agree with our findings (McCreesh & Egan, 2011). Compared to Maganaris and Baltzopoulos, our male subjects had ~0.5 cm lower MT than those studied by Maganaris and Baltzopoulos (Maganaris & Baltzopoulos, 1999). This discrepancy can be accounted for by differences in weight and height, which can explain ∼50% of the variance in skeletal mass in men and women (Janssen et al., 2000). In this line, it has been shown that body mass index (BMI) is associated with greater MT (Sanz‐Paris et al., 2021) (Usgu et al., 2021); so the higher BMI of the subjects analyzed by Maganaris and Baltzopoulos could explain the slightly higher TA MT reported in their study. Another factor that could explain differences in MT between studies relies on the procedure used to measure TA. In the present investigation, we applied the methods described by Muraoka et al. (2003), which seemed the most appropriate given that it allowed for measuring MT in the TA with small variability.

In terms of PA, our results are similar to previously published cadaveric (Sopher et al., 2017) and living human studies (Maganaris & Baltzopoulos, 1999). Although there are published data on FL change of the human TA during walking (Chleboun et al., 2007) and isometric contractions (Raiteri et al., 2016) in healthy subjects, FL data of healthy people at rest is limited (Maganaris & Baltzopoulos, 1999).

From a functional perspective, the FL should be considered conjointly with the tendon length because biomechanical properties vary depending on the tendon‐muscle fiber length ratio r_TFL,_ defined as (tendon + aponeurosis)/(fascicle length) (Morl et al., 2016; Siebert et al., 2017). For example, muscles that act as springs in bouncing gates and contribute to energy conservation have long series elastic components (SEC; aponeurosis and tendons) and short muscle fibers, resulting in high r_TFL_ (Siebert et al., 2017). This is the case of the plantaris muscle of the wallaby (r_TFL_ = 18.7) (Biewener, 1998). In contrast, muscles with motor function have a comparably short SEC and long muscle fibers like the pigeon pectoralis (r_TFL_ = 0.4) (Biewener, 1998). Since the SEC was not measured in the present investigation, it was not possible to determine precisely the r_TFL_. Nevertheless, we have done some estimations based on anatomical reports (Herbert et al., 2002). For example, the former study reported a TA's distal tendon of 230 ± 31 mm, with FL of 39 ± 8 mm for TA. However, Herbert et al. did not measure the aponeurosis. It should be noted that these FLs are shorter than ours, likely due to differences in the measurement protocol (differences in the leg's position and degree of ankle flexion). Using these data, the TA r_TFL_ should lie close to 5.9 (or higher after including the aponeurosis in the nominator). This result would correspond to a high r_TFL_, meaning that the TA is a muscle with long SEC and short muscle fibers (i.e., spring‐like behavior) (Lai et al., 2019; Siebert et al., 2017). Further studies would be needed to compute accurate in vivo r_TFL_ values for human TA, accounting for human variation and the potential effects of age, height, gender, body composition, and physical activity level.

### 4.2 Sex differences in tibialis anterior muscle architecture

The present investigation reports for the first time an analysis of the TA architectural sex differences in young, physically active humans. Females have a lower body size than males, which partly explains the smaller muscle mass of females. However, apart from body size, the anabolic effect of testosterone on muscle fibers and the higher proportion of type I fibers in females, which have lower cross‐sectional area than type II fibers, account for the sex dimorphism in muscle mass in humans (Simoneau & Bouchard, 1989). As expected, males displayed greater absolute values for MT in both regions of the TA and greater PA in the superficial region, when sex differences in body size are disregarded. However, after accounting for LLM and shank length, it became clear that females had longer fascicles than males in both regions and lower PA and MT in the superficial region.

Sex differences have been reported for other anatomical features of the lower extremity (Shultz et al., 2008). For example, there are sex differences in the Q angle, i.e., the angle between the quadriceps muscle and the patellar tendon, the distal end of the attachment site on the tibia of the TA, and the shape of the tibia. Thus, the variations in TA's attachment site suggest differences in TA's function and muscle architecture, affecting gait movement and the frequency of lower leg disorders such as chronic exertional compartment syndrome (Kimata et al., 2022). In terms of Q angle, normative Q angle values establish 11° for men and 16° for women (Horton & Hall, 1989). An increment in Q angle over these normative values would cause external rotation of the leg, while the opposite would cause internal rotation. In this regard, a higher Q angle in males has been associated with decreased isokinetic knee strength, power output, and torque (Sac & Tasmektepligil, 2018). Since it is known that joint angular rotation affects muscle architecture (Karamanidis et al., 2005), this could cause different sex‐specific muscle adaptations. However, there is controversy about this topic since the accepted, though unproven, explanation until 2005 was that women have greater Q angle than men due to a wider pelvis. However, in 2005 a group of researchers showed that this angle is similar (~2° difference) in men and women (Grelsamer et al., 2005), although contradictory results have also been found showing higher Q angle in women (Mitani, 2017). Nevertheless, caution should be taken since the quadriceps angle is highly sensitive to errors in the definition of the center of the patella and tibial tuberosity. As exposed by some authors, these centers need to be defined with an accuracy of less than 2 mm if the error in the quadriceps angle is to remain below 5° (France & Nester, 2001). Our present investigation does not allow us to ascertain whether the Q angle could contribute to explaining the small sex differences in TA FL and PA observed here.

### 4.3 Fascicle length has a suppressive effect on the pennation angle

Our causal mediation analysis highlighted a suppressive effect of FL on PA, suggesting that if the FL does not increase in length concomitantly with MT, the PA should increase around half a degree. This analysis is a method to dissect the total effect of treatment into direct and indirect effects. The indirect effect is transmitted via a mediator to the outcome. Mediation analyses are inherently causal because the mediation question sets out to explain a mechanism through which the exposure causally operates to affect the outcome. Mediation analysis is, therefore, an important statistical tool for gaining insight into the mechanisms of exposure‐outcome effects (MacKinnon, 2012). We have been able to model the relationship between these architectural variables and know how they change concerning each other by analyzing interindividual's differences in a large sample of human beings.

Some authors have found that MT and PA in resistance‐trained individuals are larger than in untrained individuals but with no differences regarding FL. These authors indicated that FL was not associated with muscle size, suggesting that FL would not increase with resistance training (Fukutani & Kurihara, 2015). Nevertheless, FL may increase with exercise training depending on the predominant type of muscle contraction (eccentric/concentric) (Franchi et al., 2016). The fact that certain types of exercise training may elicit an increase in MT and FL concurs with our causal mediation model.

The relationship between MT, FL, and PA is not always direct or causal and requires further explanation. As previously explained, an increase in MT with resistance training (Franchi et al., 2018) does not require a direct increase in the other architectural features (Franchi et al., 2016; Fukutani & Kurihara, 2015). An increase in PA allows an increase in the physiological cross‐sectional area and, thereby, maximal force‐generating capacity (Aagaard et al., 2001; Kawakami et al., 1993). However, with an increase in PA, less force from each fiber is transmitted along the line of action of the muscles (Azizi et al., 2008; Kawakami, 2005). Nevertheless, despite a less efficient transfer of force per muscle fiber, a greater PA allows for more muscle fibers to attach to the tendon as compared to a fusiform muscle (Gans & de Vree, 1987) or an increase of the amount of myofiber inside each fiber (increased fiber cross‐sectional area) allowing for the production of more force.

In summary, our causal mediation model identified a suppressive effect of FL on PA, which is in line with some authors suggesting that increments in MT are not always aligned with increments in FL or PA.

### 4.4 Strengths, limitations, and future directions

The main strengths of this study are the large number of participants analyzed, the inclusion of males and females of similar age and levels of physical activity, the use of modern ultrasound equipment, the assessment of the lean mass of the extremities to account for the effects of body size and the strict identification of anatomical landmarks and standardized procedures for image analysis. This study has also limitations, which relate to its cross‐sectional design and the fact that the architectural analysis was limited to specific regions of the muscle. However, our measurement methodology followed the latest guidelines and recommendations for musculoskeletal ultrasound (Fodor et al., 2022), and followed the methodological recommendations made by preceding researchers for TA measurement (Maganaris & Baltzopoulos, 1999; Muraoka et al., 2003).

Fascicle length and PA are major constituents of muscle architecture, and they largely determine the function and shape of the muscle, but current 2D techniques limit their precise measurement. Future studies should identify FL and PA in the TA using state‐of‐the‐art‐3D techniques such as diffusion tensor imaging (Oudeman et al., 2016; Schenk et al., 2013) to better understand the interaction of muscle with surrounding tissue and external forces (Siebert et al., 2014; Yucesoy et al., 2003). Moreover, differences in muscle architecture in the TA's superficial and deep unipennate regions may impact architectural gearing. This concept refers to the relative arrangement of muscle fibers, tendons, and aponeuroses in relation to the joint axis of rotation (Azizi & Brainerd, 2007). For example, differences in FL and PA can affect the torque‐generating capability of the muscle and its ability to produce force at different joint angles. Differences in tendon length and stiffness can impact force transmission to the bones and joints, movement efficiency, and performance. The sexual dimorphism in TA architecture could explain a lower torque‐generating capacity in females than in males when considering only MT. However, the latter could be compensated by the lower PA of females. Lastly, architectural gearing is likely to vary between different regions of the TA, contributing to this muscle's anatomical and functional complexity.

### 4.5 Conclusion

In summary, real‐time ultrasonography showed that the tibialis anterior is, two‐dimensionally, a nonsymmetrical bipennate muscle at rest in terms of muscle architecture. Moreover, a suppressive effect of fascicle length on pennation angle was identified, suggesting that increments in muscle thickness are not always aligned with increments in fascicle length or pennation angle. Small sex differences exist in tibialis anterior architecture, most of which remain after accounting for the leg lean mass and shank length.

## AUTHOR CONTRIBUTIONS

The contribution of the authors are as follows: SMR, JSM, and JALC contributed to the conception and design of the study and drafted the manuscript; JSM collected the ultrasound data and supervised all analysis; VGA and SCR helped with data collection; JJGH performed the statistical analysis and contributed to the interpretation of the findings; all coauthors critically evaluated and contributed to the manuscript. All authors have approved the final version of the manuscript.

## Supporting information


**Data S1.** Supporting Information

## Data Availability

The data that support the findings of this study are available from the corresponding author upon reasonable request.

## References

[joa13864-bib-0001] Aagaard, P. , Andersen, J.L. , Dyhre‐Poulsen, P. , Leffers, A.M. , Wagner, A. , Magnusson, S.P. et al. (2001) A mechanism for increased contractile strength of human pennate muscle in response to strength training: changes in muscle architecture. The Journal of Physiology, 534, 613–623. 11454977 10.1111/j.1469-7793.2001.t01-1-00613.x PMC2278719

[joa13864-bib-0002] Alexander, R.M. (1975) The dimensions of knee and ankle muscles and the forces they exert. Journal of Human Movement Studies, 1, 115–123.

[joa13864-bib-0003] Azizi, E. & Brainerd, E.L. (2007) Architectural gear ratio and muscle fiber strain homogeneity in segmented musculature. Journal of Experimental Zoology. Part A, Ecological Genetics and Physiology, 307, 145–155. 17397068 10.1002/jez.a.358

[joa13864-bib-0004] Azizi, E. , Brainerd, E.L. & Roberts, T.J. (2008) Variable gearing in pennate muscles. Proceedings of the National Academy of Sciences of the United States of America, 105, 1745–1750. 18230734 10.1073/pnas.0709212105 PMC2234215

[joa13864-bib-0005] Azizi, E. & Deslauriers, A.R. (2014) Regional heterogeneity in muscle fiber strain: the role of fiber architecture. Frontiers in Physiology, 5, 303. 25161626 10.3389/fphys.2014.00303 PMC4129366

[joa13864-bib-0006] Biewener, A.A. (1998) Muscle function in vivo: a comparison of muscles used for elastic energy savings versus muscles used to generate mechanical power1. American Zoologist, 38, 703–717.

[joa13864-bib-0007] Bland, D.C. , Prosser, L.A. , Bellini, L.A. , Alter, K.E. & Damiano, D.L. (2011) Tibialis anterior architecture, strength, and gait in individuals with cerebral palsy. Muscle & Nerve, 44, 509–517. 21755515 10.1002/mus.22098 PMC3175274

[joa13864-bib-0008] Blazevich, A.J. , Gill, N.D. & Zhou, S. (2006) Intra‐ and intermuscular variation in human quadriceps femoris architecture assessed in vivo. Journal of Anatomy, 209, 289–310. 16928199 10.1111/j.1469-7580.2006.00619.x PMC2100333

[joa13864-bib-0009] Calbet, J.A.L. , Ponce‐Gonzalez, J.G. , Calle‐Herrero, J. , Perez‐Suarez, I. , Martin‐Rincon, M. , Santana, A. et al. (2017) Exercise preserves lean mass and performance during severe energy deficit: the role of exercise volume and dietary protein content. Frontiers in Physiology, 8, 483. 28790922 10.3389/fphys.2017.00483 PMC5522839

[joa13864-bib-0010] Chleboun, G.S. , Busic, A.B. , Graham, K.K. & Stuckey, H.A. (2007) Fascicle length change of the human tibialis anterior and vastus lateralis during walking. The Journal of Orthopaedic and Sports Physical Therapy, 37, 372–379. 17710906 10.2519/jospt.2007.2440

[joa13864-bib-0011] Di Giulio, I. , Maganaris, C.N. , Baltzopoulos, V. & Loram, I.D. (2009) The proprioceptive and agonist roles of gastrocnemius, soleus and tibialis anterior muscles in maintaining human upright posture. The Journal of Physiology, 587, 2399–2416. 19289550 10.1113/jphysiol.2009.168690 PMC2697307

[joa13864-bib-0012] Eng, C.M. , Azizi, E. & Roberts, T.J. (2018) Structural determinants of muscle gearing during dynamic contractions. Integrative and Comparative Biology, 58, 207–218. 29889236 10.1093/icb/icy054 PMC6104701

[joa13864-bib-0013] Fodor, D. , Rodriguez‐Garcia, S.C. , Cantisani, V. , Hammer, H.B. , Hartung, W. , Klauser, A. et al. (2022) The EFSUMB guidelines and recommendations for musculoskeletal ultrasound ‐ part I: Extraarticular pathologies. Ultraschall in der Medizin, 43, 34–57. 34479372 10.1055/a-1562-1455

[joa13864-bib-0014] France, L. & Nester, C. (2001) Effect of errors in the identification of anatomical landmarks on the accuracy of Q angle values. Clinical Biomechanics (Bristol, Avon), 16, 710–713. 11535354 10.1016/s0268-0033(01)00045-6

[joa13864-bib-0015] Franchi, M.V. , Atherton, P.J. , Maganaris, C.N. & Narici, M.V. (2016) Fascicle length does increase in response to longitudinal resistance training and in a contraction‐mode specific manner. Springerplus, 5, 94. 26848434 10.1186/s40064-015-1548-8 PMC4731380

[joa13864-bib-0016] Franchi, M.V. , Fitze, D.P. , Raiteri, B.J. , Hahn, D. & Sporri, J. (2020) Ultrasound‐derived biceps Femoris long head fascicle length: extrapolation pitfalls. Medicine and Science in Sports and Exercise, 52, 233–243. 31403609 10.1249/MSS.0000000000002123

[joa13864-bib-0017] Franchi, M.V. , Raiteri, B.J. , Longo, S. , Sinha, S. , Narici, M.V. & Csapo, R. (2018) Muscle architecture assessment: strengths, shortcomings and new Frontiers of in vivo imaging techniques. Ultrasound in Medicine & Biology, 44, 2492–2504. 30185385 10.1016/j.ultrasmedbio.2018.07.010

[joa13864-bib-0018] Freiman, J.A. , Chalmers, T.C. , Smith, H.A. & Kuebler, R.R. (2019) The importance of beta, the type II error, and sample size in the design and interpretation of the randomized controlled trial: survey of two sets of “negative” trials. In: Medical uses of statistics. Boca Raton, FL: CRC Press, pp. 357–389.

[joa13864-bib-0019] Fukutani, A. & Kurihara, T. (2015) Comparison of the muscle fascicle length between resistance‐trained and untrained individuals: cross‐sectional observation. Springerplus, 4, 341. 26185743 10.1186/s40064-015-1133-1 PMC4499036

[joa13864-bib-0020] Gans, C. & de Vree, F. (1987) Functional bases of fiber length and angulation in muscle. Journal of Morphology, 192, 63–85. 3455200 10.1002/jmor.1051920106

[joa13864-bib-0021] Grelsamer, R.P. , Dubey, A. & Weinstein, C.H. (2005) Men and women have similar Q angles: a clinical and trigonometric evaluation. Journal of Bone and Joint Surgery, 87, 1498–1501. 10.1302/0301-620X.87B11.16485 16260666

[joa13864-bib-0022] Herbert, R.D. , Moseley, A.M. , Butler, J.E. & Gandevia, S.C. (2002) Change in length of relaxed muscle fascicles and tendons with knee and ankle movement in humans. The Journal of Physiology, 539, 637–645. 11882694 10.1113/jphysiol.2001.012756 PMC2290150

[joa13864-bib-0023] Horton, M.G. & Hall, T.L. (1989) Quadriceps femoris muscle angle: normal values and relationships with gender and selected skeletal measures. Physical Therapy, 69, 897–901. 2813517 10.1093/ptj/69.11.897

[joa13864-bib-0024] Ihnatsenka, B. & Boezaart, A.P. (2010) Ultrasound: basic understanding and learning the language. International Journal of Shoulder Surgery, 4, 55–62. 21472065 10.4103/0973-6042.76960 PMC3063344

[joa13864-bib-0025] Imai, K. , Keele, L. & Tingley, D. (2010) A general approach to causal mediation analysis. Psychological Methods, 15, 309–334. 20954780 10.1037/a0020761

[joa13864-bib-0026] Janssen, I. , Heymsfield, S.B. , Wang, Z.M. & Ross, R. (2000) Skeletal muscle mass and distribution in 468 men and women aged 18‐88 yr. Journal of Applied Physiology, 89, 81–88. 10904038 10.1152/jappl.2000.89.1.81

[joa13864-bib-0027] Karamanidis, K. , Stafilidis, S. , DeMonte, G. , Morey‐Klapsing, G. , Bruggemann, G.P. & Arampatzis, A. (2005) Inevitable joint angular rotation affects muscle architecture during isometric contraction. Journal of Electromyography and Kinesiology, 15, 608–616. 16179198 10.1016/j.jelekin.2005.02.001

[joa13864-bib-0028] Kawakami, Y. (2005) The effects of strength training on muscle architecture in humans. International Journal of Sport and Health Science, 3, 208–217.

[joa13864-bib-0029] Kawakami, Y. , Abe, T. & Fukunaga, T. (1993) Muscle‐fiber pennation angles are greater in hypertrophied than in normal muscles. Journal of Applied Physiology, 74, 2740–2744. 8365975 10.1152/jappl.1993.74.6.2740

[joa13864-bib-0030] Keith, L. , Moore, A. & Agur, A. (2006) Clinically oriented anatomy. Philadelphia, PA: Lippincott Williams & Wilkins.

[joa13864-bib-0031] Kimata, K. , Otsuka, S. , Yokota, H. , Shan, X. , Hatayama, N. & Naito, M. (2022) Relationship between attachment site of tibialis anterior muscle and shape of tibia: anatomical study of cadavers. Journal of Foot and Ankle Research, 15, 54. 35821059 10.1186/s13047-022-00559-y PMC9277928

[joa13864-bib-0032] Koo, T.K. & Li, M.Y. (2016) A guideline of selecting and reporting Intraclass correlation coefficients for reliability research. Journal of Chiropractic Medicine, 15, 155–163. 27330520 10.1016/j.jcm.2016.02.012 PMC4913118

[joa13864-bib-0033] Kunimasa, Y. , Sano, K. , Oda, T. , Nicol, C. , Komi, P.V. , Locatelli, E. et al. (2014) Specific muscle‐tendon architecture in elite Kenyan distance runners. Scandinavian Journal of Medicine & Science in Sports, 24, e269–e274. 26207267 10.1111/sms.12161

[joa13864-bib-0034] Kwah, L.K. , Pinto, R.Z. , Diong, J. & Herbert, R.D. (2013) Reliability and validity of ultrasound measurements of muscle fascicle length and pennation in humans: a systematic review. Journal of Applied Physiology, 114, 761–769. 23305989 10.1152/japplphysiol.01430.2011

[joa13864-bib-0035] Lai, A.K.M. , Biewener, A.A. & Wakeling, J.M. (2019) Muscle‐specific indices to characterise the functional behaviour of human lower‐limb muscles during locomotion. Journal of Biomechanics, 89, 134–138. 31036379 10.1016/j.jbiomech.2019.04.027 PMC6512870

[joa13864-bib-0036] Lee, H. , Cashin, A.G. , Lamb, S.E. , Hopewell, S. , Vansteelandt, S. , VanderWeele, T.J. et al. (2021) A guideline for reporting mediation analyses of randomized trials and observational studies: the AGReMA statement. JAMA, 326, 1045–1056. 34546296 10.1001/jama.2021.14075 PMC8974292

[joa13864-bib-0037] Lenth, R. , Singmann, H. , Love, J. , Buerkner, P. & Herve, M. (2018) Emmeans: estimated marginal means, aka least‐squares means. R Package Version 1.3.

[joa13864-bib-0038] MacKinnon, D.P. (2012) Introduction to statistical mediation analysis. London, UK: Routledge.

[joa13864-bib-0039] Maganaris, C.N. & Baltzopoulos, V. (1999) Predictability of in vivo changes in pennation angle of human tibialis anterior muscle from rest to maximum isometric dorsiflexion. European Journal of Applied Physiology and Occupational Physiology, 79, 294–297. 10048637 10.1007/s004210050510

[joa13864-bib-0040] Maharaj, J.N. , Cresswell, A.G. & Lichtwark, G.A. (2019) Tibialis anterior tendinous tissue plays a key role in energy absorption during human walking. The Journal of Experimental Biology, 222, jeb191247. 31064856 10.1242/jeb.191247

[joa13864-bib-0041] McCreesh, K. & Egan, S. (2011) Ultrasound measurement of the size of the anterior tibial muscle group: the effect of exercise and leg dominance. Sports Medicine, Arthroscopy, Rehabilitation, Therapy & Technology: SMARTT, 3, 18. 10.1186/1758-2555-3-18 PMC3180254 21914209

[joa13864-bib-0042] Mitani, Y. (2017) Gender‐related differences in lower limb alignment, range of joint motion, and the incidence of sports injuries in Japanese university athletes. Journal of Physical Therapy Science, 29, 12–15. 28210029 10.1589/jpts.29.12 PMC5300795

[joa13864-bib-0043] Morl, F. , Siebert, T. & Haufle, D. (2016) Contraction dynamics and function of the muscle‐tendon complex depend on the muscle fibre‐tendon length ratio: a simulation study. Biomechanics and Modeling in Mechanobiology, 15, 245–258. 26038176 10.1007/s10237-015-0688-7

[joa13864-bib-0044] Muraoka, T. , Muramatsu, T. , Kanehisa, H. & Fukunaga, T. (2003) Transverse strain of aponeurosis in human tibialis anterior muscle at rest and during contraction at different joint angles. Journal of Applied Biomechanics, 19, 39–48.

[joa13864-bib-0045] Nuzzo, J.L. , Finn, H.T. & Herbert, R.D. (2019) Causal mediation analysis could resolve whether training‐induced increases in muscle strength are mediated by muscle hypertrophy. Sports Medicine, 49, 1309–1315. 31161403 10.1007/s40279-019-01131-8

[joa13864-bib-0046] Oudeman, J. , Mazzoli, V. , Marra, M.A. , Nicolay, K. , Maas, M. , Verdonschot, N. et al. (2016) A novel diffusion‐tensor MRI approach for skeletal muscle fascicle length measurements. Physiological Reports, 4, e13012. 28003562 10.14814/phy2.13012 PMC5210383

[joa13864-bib-0047] Perry, M.C. , Carville, S.F. , Smith, I.C. , Rutherford, O.M. & Newham, D.J. (2007) Strength, power output and symmetry of leg muscles: effect of age and history of falling. European Journal of Applied Physiology, 100, 553–561. 16847676 10.1007/s00421-006-0247-0

[joa13864-bib-0048] Potier, T.G. , Alexander, C.M. & Seynnes, O.R. (2009) Effects of eccentric strength training on biceps femoris muscle architecture and knee joint range of movement. European Journal of Applied Physiology, 105, 939–944. 19271232 10.1007/s00421-008-0980-7

[joa13864-bib-0049] Powell, P.L. , Roy, R.R. , Kanim, P. , Bello, M.A. & Edgerton, V.R. (1984) Predictability of skeletal muscle tension from architectural determinations in Guinea pig hindlimbs. Journal of Applied Physiology: Respiratory, Environmental and Exercise Physiology, 57, 1715–1721. 6511546 10.1152/jappl.1984.57.6.1715

[joa13864-bib-0050] Raiteri, B.J. , Cresswell, A.G. & Lichtwark, G.A. (2016) Three‐dimensional geometrical changes of the human tibialis anterior muscle and its central aponeurosis measured with three‐dimensional ultrasound during isometric contractions. PeerJ, 4, e2260. 27547566 10.7717/peerj.2260 PMC4974924

[joa13864-bib-0051] Sac, A. & Tasmektepligil, M.Y. (2018) Correlation between the Q angle and the isokinetic knee strength and muscle activity. Turkish Journal of Physical Medicine and Rehabilitation, 64, 308–313. 31453527 10.5606/tftrd.2018.2366 PMC6648034

[joa13864-bib-0052] Sanz‐Paris, A. , Gonzalez‐Fernandez, M. , Hueso‐Del Rio, L.E. et al. (2021) Muscle thickness and echogenicity measured by ultrasound could detect local sarcopenia and malnutrition in older patients hospitalized for hip fracture. Nutrients, 13, 2401. 34371911 10.3390/nu13072401 PMC8308882

[joa13864-bib-0053] Schenk, P. , Siebert, T. , Hiepe, P. , Güllmar, D. , Reichenbach, J.R. , Wick, C. et al. (2013) Determination of three‐dimensional muscle architectures: validation of the DTI‐based fiber tractography method by manual digitization. Journal of Anatomy, 223, 61–68. 23678961 10.1111/joa.12062 PMC4487763

[joa13864-bib-0054] Shultz, S.J. , Nguyen, A.D. & Schmitz, R.J. (2008) Differences in lower extremity anatomical and postural characteristics in males and females between maturation groups. The Journal of Orthopaedic and Sports Physical Therapy, 38, 137–149. 18383647 10.2519/jospt.2008.2645

[joa13864-bib-0055] Siebert, T. , Till, O. & Blickhan, R. (2014) Work partitioning of transversally loaded muscle: experimentation and simulation. Computer Methods in Biomechanics and Biomedical Engineering, 17, 217–229. 22515574 10.1080/10255842.2012.675056

[joa13864-bib-0056] Siebert, T. , Tomalka, A. , Stutzig, N. , Leichsenring, K. & Bol, M. (2017) Changes in three‐dimensional muscle structure of rabbit gastrocnemius, flexor digitorum longus, and tibialis anterior during growth. Journal of the Mechanical Behavior of Biomedical Materials, 74, 507–519. 28778781 10.1016/j.jmbbm.2017.07.045

[joa13864-bib-0057] Simoneau, J.A. & Bouchard, C. (1989) Human variation in skeletal muscle fiber‐type proportion and enzyme activities. The American Journal of Physiology, 257, E567–E572. 2529775 10.1152/ajpendo.1989.257.4.E567

[joa13864-bib-0058] Sopher, R.S. , Amis, A.A. , Davies, D.C. & Jeffers, J.R. (2017) The influence of muscle pennation angle and cross‐sectional area on contact forces in the ankle joint. Journal of Strain Analysis for Engineering Design, 52, 12–23. 29805194 10.1177/0309324716669250 PMC5952297

[joa13864-bib-0059] Tingley, D. , Yamamoto, T. , Hirose, K. , Keele, L. & Imai, K. (2014) Mediation: R package for causal mediation analysis.

[joa13864-bib-0060] Usgu, S. , Ramazanoglu, E. & Yakut, Y. (2021) The relation of body mass index to muscular viscoelastic properties in Normal and overweight individuals. Medicina, 57, 1022. 34684059 10.3390/medicina57101022 PMC8537384

[joa13864-bib-0061] Yucesoy, C.A. , Koopman, B.H. , Baan, G.C. , Grootenboer, H.J. & Huijing, P.A. (2003) Extramuscular myofascial force transmission: experiments and finite element modeling. Archives of Physiology and Biochemistry, 111, 377–388. 15764078 10.3109/13813450312331337630

[joa13864-bib-0062] Zielinska, N. , Tubbs, R.S. , Paulsen, F. , Szewczyk, B. , Podgórski, M. , Borowski, A. et al. (2021) Anatomical variations of the tibialis anterior tendon insertion: an updated and comprehensive review. Journal of Clinical Medicine, 10, 3684. 34441980 10.3390/jcm10163684 PMC8396864

